# Synesthesia: a colorful word with a touching sound?

**DOI:** 10.3389/fpsyg.2013.00763

**Published:** 2013-10-22

**Authors:** Myrto I. Mylopoulos, Tony Ro

**Affiliations:** ^1^Philosophy Program, The Graduate Center, City University of New York, New York, NY, USA; ^2^Department of Psychology, The City College and Graduate Center, Program in Cognitive Neuroscience, City University of New YorkNew York, NY, USA

**Keywords:** synesthesia, sensation, perception, imagery, memory

## Abstract

Synesthesia is a fairly common condition in which individuals experience atypical responses (such as color experiences) in association with certain types of stimuli (such as non-colored letters). Although synesthesia has been described for centuries, only very recently has there been an explosive growth of systematic scientific examinations of this condition. In this article, we review and critically evaluate current methods for both assessing synesthesia and examining its psychological basis, including the “test-retest” procedure, online battery assessments, and behavioral experiments. We highlight the limitations of these methods for understanding the nature of this complex condition and propose potential solutions to address some of these limitations. We also provide a set of markers that aid in distinguishing synesthesia from other closely related psychological phenomena.

## Introduction

Synesthesia is a condition in which individuals experience atypical responses to certain types of stimuli, in addition to the typical responses elicited by those stimuli. For example, a synesthete may perceive tastes when seeing certain shapes or might perceive colors when seeing achromatic letters. Synesthesia comes in many forms, covering a wide range of sensory interactions both cross-modally and within a single modality^1^.

Over the years, a variety of research programs have emerged to better understand this condition. Some research on synesthesia is focused on determining how and why synesthetic associations are developed or acquired, and the patterns, if any, that govern them (Watson et al., 2010, 2012; Witthoft and Winawer, 2013). Other research has been devoted to determining the nature and characteristic features of synesthetic associations, raising questions, for example, regarding the types of stimuli that can trigger synesthetic responses (Ramachandran et al., 2002). But perhaps the most pressing questions pertaining to synesthetic associations concern their psychological *kind*. Are they genuinely perceptual, as many claim? In other words, are synesthetic responses the outputs of sensory modalities, exhibiting features that correspond to the sensory qualities of stimuli such as color, shape, and sound? Or are they purely cognitive responses? Are they mnemonic associations? Or are they some combination thereof?

While significant steps forward have been taken in synesthesia research in the past couple of decades, there is still further to go when it comes to establishing conclusive answers to these questions using objective measures. Some of the biggest strides are being made using neuroimaging techniques that are helping to reveal the neural basis of synesthesia. While we think these techniques are particularly promising for understanding synesthesia, in this short review, we focus instead on surveying and critically evaluating popular behavioral strategies for assessing and understanding synesthesia. We highlight the limitations of these strategies when it comes to both accurately assessing cases of synesthesia and examining the nature of the responses involved, and we propose potential solutions to some of these limitations along the way.

## Assessing synesthesia

In order to assess or diagnose a psychological condition, one must, of course, know what to look for. Although much about synesthesia is still unknown, and novel forms and varieties of the condition may manifest themselves, it will nonetheless be useful to highlight some characteristics of synesthesia that serve to distinguish it from other perceptual phenomena, such as visual imagery and certain forms of imagistic memory. There are three such characteristic features: (1) automaticity, (2) reliability, and (3) consistency^2^. First, there is ample evidence that synesthetic associations are *automatic* in nature (Lupiáñez and Callejas, 2006; Jarick et al., 2011). They are typically produced outside the intentional control of the individual and cannot be directly inhibited. The automaticity of synesthesia helps to distinguish it from paradigm cases of mental imagery. While hearing a certain sound may lead one to imagine certain scenes or colors, for example, such visual imagery is typically under a significant degree of intentional control. One can usually start or stop imagining something at will. This is not to deny that synesthetic responses have qualities—for example, shapes and colors—that are similar to or even the same as those exhibited by mental imagery. However, synesthetic responses are, at the very least, distinguishable from mental imagery in virtue of their automaticity.

Second, it is typically the case that synesthetes *reliably* experience synesthetic responses when presented with triggering stimuli. When synesthetes come into perceptual contact with the triggering stimulus, their responses will be induced. These responses are not transient or inconsistent, though they may sometimes be when induced neuropharmacologically, for example, by psychoactive, hallucinogenic substances^3^. Indeed, synesthesia is often present from early childhood onwards (Cytowic, 2002). This helps to distinguish the condition from ordinary associations grounded in memory. One sometimes has vivid mnemonic imagery associated with specific smells or sounds, for example, and these memories may sometimes even arise automatically. But it is uncommon for these to be reliably generated and persistent throughout an individual's life in the way that synesthetic responses are reported to be.

Finally, although there is variability across synesthetes, synesthetic associations within an individual appear to remain relatively *consistent* over time in that the same types of stimuli (e.g., specific auditory tones) tend to elicit the same types of synesthetic responses (e.g., specific colors)^4^,^5^ (Dixon et al., 2000). This feature, too, helps to distinguish synesthetic associations from ordinary mental imagery, which displays more flexible associations. But as Simner (2012) argues, consistency may not be central to synesthesia in the way that many have supposed. The problem is that many of the tests used to assess synesthesia, as we will see, treat consistency as the main measure of synesthetic association. As a result, the synesthetes who are examined in the psychological literature are all those that exhibit consistency in their associations. This bias may have created an inflated sense of how common this characteristic really is among synesthetic associations, to the point where it has become a defining feature of the condition. We therefore restrict ourselves to the following claim: when consistency is present, this provides evidence in favor of the relevant association being a synesthetic one. However, when consistency is absent, this is *not* evidence *against* the relevant association being a synesthetic one.

Given that assessment strategies are used as diagnostic tools for establishing whether a given individual is a synesthete, it is important that they are able to establish that certain reported associations exhibit each of these characteristics and perhaps others that remain to be identified. One of the more widely accepted measures for determining whether one has synesthesia is the test-retest procedure, known as the “test of genuineness” (TOG) among synesthesia researchers. The TOG is considered by some to be the “gold standard” of synesthesia assessment. In this procedure, synesthetes are asked to indicate, either through verbal report or color swatch matching, the character of their synesthetic responses to certain stimuli, and then they are retested—often without warning—as much as a year or more later (Baron-Cohen et al., 1987; Cytowic, 2002; Asher et al., 2006).

The rationale is that if a person has synesthesia, their consistency of responses at the retest phase will be significantly higher than those without synesthesia, who are told to simply assign associations to the same set of stimuli. For example, Baron-Cohen et al. (1993) found that 92.3% of the reported synesthetes they tested gave consistent responses when they were retested one year later without warning, whereas this was true of only 37.6% of control subjects who were tested one week later with warning. This finding, and others like it, are made all the more impressive by the fact that synesthetic responses are often very precise, for example, sometimes with highly specific hues and shades in grapheme-color synesthesia (Eagleman et al., 2007). In cases where there is a high consistency of responses, therefore, it is likely the result of a stable association. The TOG can therefore provide strong evidence that reported associations possess one of the common characteristics of synesthesia. If one fails the TOG, however, in line with our earlier caveats, this alone is not evidence against a reported association being a synesthetic one, since it may simply be that the association is not consistent.

**Figure 1:**
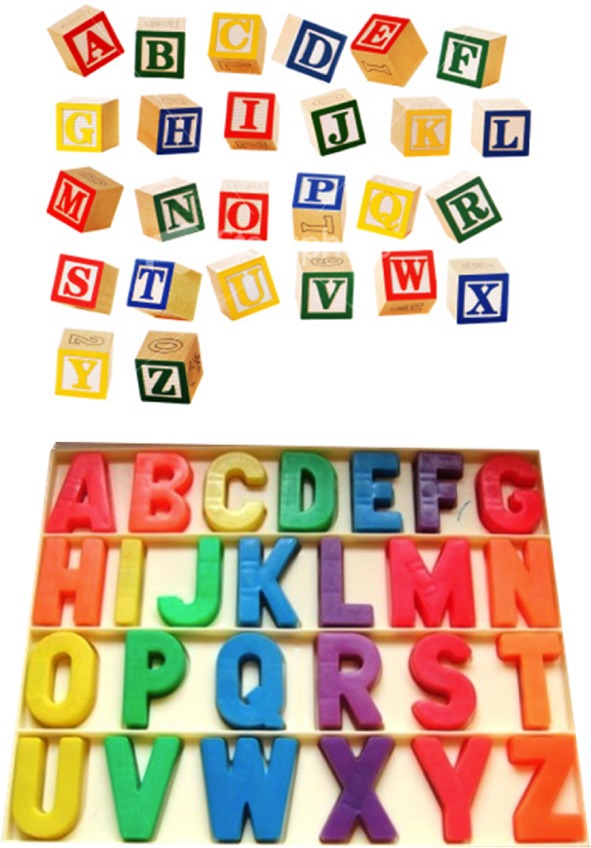
**Letter-color associations may have been acquired early on in development through common associations between letters and colors, as illustrated in these toys**.

The TOG faces a number of shortcomings. Response consistencies over time do not by themselves indicate whether the associations in question are automatic, or whether they are instead, in some cases, being conjured up at will. Moreover, they do not by themselves establish the reliability of the associations, as typically only two instances of each association are observed—once in the testing phase and then again in the retest phase. In addition, this procedure provides no evidence as to whether the associations are cognitive, perceptual, mnemonic, or some combination of these. It might be that some individuals who are classified as having grapheme-color synesthesia may simply have better or stronger memories overall, as it has been reported in at least one case study (Smilek et al., 2002; see also Tammet, 2009, p. 73) and in a larger group study (Radvansky et al., 2011). They may thus have robust mnemonic associations of certain colors with certain letters or numbers, though these enhanced memory effects may be modest in size (see Yaro and Ward, 2007; Rothen et al., 2012)^6^. When taking the TOG, some individuals may simply be voluntarily associating in memory rather than automatically perceiving the color red with the letter “A,” just as one may recall his or her grandmother's face when smelling freshly baked chocolate chip cookies. These associations, which may in some other cases be automatic, could have been learned early on in childhood, such as through television, books, toys, or refrigerator magnets (see Witthoft and Winawer, 2006, 2013) (Figure 1). Though voluntarily associating with superior memory is unlikely to account for all cases in which consistency is displayed, our point here is that it may be operative in enough cases that the reliability of the TOG is diminished.

We propose that this issue may possibly be addressed by adding to the TOG a further test and retest using stimuli that are not claimed by self-reported synesthetes to yield synesthetic associations, such as stimuli from a sensory modality that does not produce synesthetic responses. For example, a grapheme-color synesthete could be tested with auditory stimuli. The results of this test-retest for non-synesthetic associations could then be compared with those involving the reportedly synesthetic associations. This would control for the role that superior memory may play in generating the consistent results *within* each individual.

We also note some complications regarding the role of memory in synesthetic associations. We may distinguish among three types of sensory associations: (1) associations between a stimulus and a response that are generated by willful perceptual imagery (whether mnemonically based or not), (2) associations between a stimulus and a response that are generated by automatic perceptual imagery that are also mnemonically based, and (3) associations between a stimulus and a response that are generated by automatic perceptual imagery, but that are not mnemonically based. We are inclined not to view the first kind of association as synesthetic, since it is not automatic in character. We take the third kind of association to be very similar to, if not the same, as non-imagery based synesthetic associations. There is a question, however, about whether the second type of association is properly understood as synesthetic association, involving as it does an indirect pathway between the stimulus and response—going through mnemonic systems and then back to perceptual systems, rather than directly through perceptual systems. We leave this question open for future theorizing.

Given the subjective nature of the responses generated by synesthesia, especially problematic are online assessments or batteries for this condition (e.g., Eagleman et al., 2007). In these assessments, individuals are given a battery of tests aiming to capture any synesthetic responses the online test takers may possess. These assessments suffer from two main flaws. The first is a problem for online assessments or batteries more generally, which is that the way in which one responds on such batteries cannot be directly monitored. Thus, subjects may use inappropriate strategies, may take the battery on several occasions (see Birnbaum, 2004), may not respond consistently just to get through the assessment faster, or may use notes or visual aids to produce their consistent responses. They may also use other cues, such as spatial ones to indicate a “perceived” color on a color bar or wheel that might increase accuracy and consistency in their responses. For example, if a color bar always has the same colors in the same order from the top to the bottom of a monitor, as in some synesthesia batteries, subjects may use position on the bar relative to the monitor, as well as other potential screen landmarks, to increase their precision^7^. The inability to verify whether subjects are responding appropriately to the assessment, while a general problem with online tests, is especially problematic in the case of assessing synesthesia, where there are already difficulties in classifying the condition, and in determining how to carry these assessments out so as to capture its main subjective features. In addition, there is a bias present in such assessments since many of those who take the test believe themselves to already have synesthesia^8^.

Another problem with the online battery is that, like the TOG, it cannot establish the automaticity or reliability of synesthetic associations. It is therefore not comprehensive in the way that a serious assessment of a psychological condition must be if it is to achieve reliable results. In addition, though frequency estimates of synesthesia are not commonly based solely on such measures, any that are (e.g., Novich et al., 2011 regarding the frequency of types of synesthesia) should be interpreted cautiously.

We acknowledge that there is a benefit in using online batteries in that they allow researchers to cast a much wider net than laboratory studies and to thereby collect data on a larger pool of individuals. But we propose that, in order for the larger set of data to guide research appropriately, these tests be used in tandem with laboratory studies to validate the results collected online.

## Understanding synesthesia: psychological paradigms

In this section, we explore three commonly used paradigms for examining the character of synesthetic responses. We stress some concerns with the current state of the literature, and offer some potential remedies.

### Stroop task

Early attempts to explore the nature of synesthetic associations made use of a variation of the Stroop task (Stroop, 1935; MacLeod, 1991), in which color naming responses are substantially delayed for words that spell out incompatible colors (e.g., naming the color red when the word “GREEN” is printed or displayed in red). In this variation, synesthetes are presented with graphemes that are written in a color that is either congruent or incongruent with the synesthetic colors that they report associating with these graphemes. So, for example, if a synesthete reports associating the letter “A” with the color red, she would be presented with a red “A” in the congruent condition, and an “A” in some other color in the incongruent condition. The subjects are then asked to name aloud the color of the grapheme. Synesthetes tend to respond more slowly on incongruent trials than on congruent trials compared with controls, for whom no effect on reaction times is found (Dixon et al., 2000; Mattingley et al., 2001; Lupiáñez and Callejas, 2006).

The Stroop task is a valuable tool for establishing the automaticity of synesthetic associations. The slower reaction times on the incongruent trials are interpreted as an inability to inhibit the interfering synesthetic response—and this is a key characteristic of automatic association. As this is not something that the TOG or online battery tests probe, we propose that a more comprehensive assessment of synesthesia may combine these tests with a Stroop-like test, so that the automatic dimension of the condition may be examined. In general, a successful assessment strategy for synesthesia may need to combine multiple tests in order to get a clearer profile of the condition within any given individual.

But, as others have noted (e.g., Hubbard et al., 2005; Gheri et al., 2008), these results do not help to establish that synesthetic responses are perceptual in nature since the interference could be due to purely cognitive rather than perceptual processes. Indeed, Elias et al. (2003) used this variant of the Stroop paradigm to compare the performance of a grapheme-color synesthete and a control who was trained on semantic color-number associations using a set of specific cross-stitch knitting patterns over a period of 8 years. For example, the number 5 might indicate that one should use red thread, and would thereby be strongly associated with the color red. They found that *both* the synesthete and the semantically trained subject were slower to name colors of numbers that were incongruent with their specific associations, suggesting that interference in this Stroop paradigm need not be perceptual in nature. Colizoli et al. (2012) similarly found Stroop interference effects in a training study using non-synesthetes. In light of these results, the Stroop paradigm, like the test-retest procedure, may be inappropriate for objectively establishing the perceptual character of synesthesia, at least when used in isolation.

There are two obstacles to this conclusion, however. The first is that other training studies using non-synesthetes have not found interference effects post-training (Rothen et al., 2011; Kusnir and Thut, 2012), so further research is needed in order to determine whether interference in the Stroop paradigm can be the result of learned, non-perceptual associations. Second, the magnitude of the interference found in studies with trained non-synesthetes may be smaller than that found for synesthetes in the Stroop task, though direct comparisons between studies is difficult because of the different stimuli and scales that were used (see also Elias et al., 2003, in which the trained non-synesthete displayed more interference on one of the tasks). This suggests a more subtle difference between synesthetic automaticity and that of non-synesthetic associations. Using neuroimaging along with the Stroop task will help to establish both the perceptual nature of synesthesia, if perceptual regions of the brain such as color areas are activated, as well as the automaticity of these effects, given that the Stroop task is a reliable measure of this feature.

### Visual search tasks

Another popular paradigm used to understand synesthetic associations explores the relationship between synesthesia and attention. In a visual search task, one is presented with an array of stimuli and asked to respond to the presence or absence of a “target” stimulus that differs from the other “distractor” stimuli on the basis of some visual feature, such as color, orientation, or shape. When the target stimulus differs from the distractor stimuli with respect to just one of these features, it tends to grab attention, regardless of the number of distractors. One commonly offered explanation for this is that individual features of stimuli are processed automatically and in parallel by different parts of the visual system before they are bound together by attention, and thus seem to “pop-out” from the feature search visual array (Treisman and Gelade, 1980). Applying this to synesthesia, the rationale is that if a synesthetic color exhibits this pop-out effect during a visual search task on which the target differs from distractors on the basis of a unique feature, then it must be processed preattentively in the same way that veridical colors are in such tasks.

To test this hypothesis, Ramachandran and Hubbard (2001) conducted a pioneering study, where they briefly presented two projector synesthetes and forty controls with arrays of achromatic graphemes, where one group of graphemes formed an embedded shape (square, rectangle, triangle, or diamond). For example, the array might consist of a triangle of 2 s amongst background filled with 5s. The particular graphemes used to form the embedded shape were predicted to trigger synesthetic colors for the two synesthetes. Participants in the experiment were asked to identify the embedded shape. The two synesthetes were significantly better than controls at successfully identifying the embedded shape from a set of four options, presumably because the graphemes induced colors that popped out from the background. However, other studies seeking to replicate this result have not all been successful, and some with even larger samples of synesthetes (see Table 1).

**Table 1:** **A summary of studies testing the performance of synesthetes using visual search tasks**.

**Authors of study**	**Type of visual**	**Number of**	**Superior**
	**search task**	**synesthetes**	**performance for**
		**tested**	**synesthetes?**
Nijboer et al., 2011	Single target	9 synesthetes	No
Palmeri et al., 2002	Single target	1 synesthete	Yes
Laeng et al., 2004	Single target	1 synesthete	Yes
Edquist et al., 2006	Single target	14 synesthetes	No
Sagiv et al., 2006	Single target	2 synesthetes	No
Gheri et al., 2008	Single target	7 synesthetes	No
Ramachandran and Hubbard, 2001	Embedded figure	2 synesthetes	Yes
Hubbard et al., 2005	Embedded figure	6 synesthetes	Yes
Rothen and Meier, 2009	Embedded figure	13 synesthetes	No
Ward et al., 2010	Embedded figure	36 synesthetes	Yes
Hubbard et al., 2006	Embedded figure	1 synesthete	Yes

Studies using a more traditional visual search paradigm, in which participants are not asked to identify an embedded shape, but rather to locate a single target stimulus among distractors, have come up with mixed results. For example, Palmeri et al. (2002) found that the synesthete WO, who associates a specific color with the digit “2,” was both significantly faster than the controls at spotting a target “2” among a set of “5” s and showed a significantly smaller effect of set size on search time. Laeng et al. (2004) also found superior performance in one synesthete using the same task. However, others have not arrived at the same findings (see Table 1). Until these contradictory results across studies can be sorted out, the general conclusion that synesthetes outperform controls on visual search tasks, either the embedded figure variant or the single target version, is not warranted.

A further concern is that it is not clear that the superior performance of synesthetes, in cases where it is indeed present, is due to synesthetic responses preattentively generating the pop-out effect. Ward et al. (2010) examined this very assumption. They conducted a study with 36 synesthetes, using Ramachandran and Hubbard's embedded-figure task, but this time including an assessment of the synesthetes' self-reports during the experiment. They found that, although synesthetes did tend to outperform the controls, most synesthetes reported that they did not experience synesthetic responses across the entire array during the task. Perhaps more importantly, the synesthetes that did experience synesthetic responses reported them as appearing piecemeal, rather than all at once, suggesting that these experiences depend on attention and perhaps other higher-order processes, and do not therefore pop-out like veridical colors in such tasks. As an example of a typical participant's report, Ward et al. offer this revealing quotation: “I definitely do NOT see all the colors in one go. I have to attend to the symbols/shapes or process them in some way, and then it has a color attributed to it. It's not like I could be looking somewhere else, and in the corner I see a shape made out of shapes of one color.”

This verbal report is corroborated by a recent study by Nijboer et al. (2011) using a visual search task on which synesthetes and controls had to spot a single target digit among a set of distractor digits, for example, a “2” among a set of “5” s. An interesting feature of their experiment is that participants were required to make just a single, direct eye movement to the target, rather than being allowed to wander their eyes around the array. The target and distractors were either all gray (achromatic condition) or all colored. The target stimulus was always a different color from the distractors. Nijboer et al. found that synesthetes performed comparably to controls in both the chromatic and achromatic conditions. Importantly, accuracy decreased with increases in set size in the achromatic condition for both the synesthetes and the controls, indicating that no pop-out effect occurred for either group. Furthermore, there was no effect of set size on accuracy in the chromatic condition, indicating that pop-out *did* occur in this condition for both groups. This evidence seems to cast into doubt the claim that synesthetic responses are generated preattentively.

Perhaps a better way to assess whether attention is necessary for synesthetic responses is to determine whether perceptual load (e.g., Lavie and Tsal, 1994; Lavie, 1995) affects the occurrence of these responses. Perceptual load refers to the amount of perceptual information in a stimulus or a set of stimuli, with more attentional resources being required for processing when perceptual load is high. In particular, if attention is required for a synesthetic response to occur, then these responses should be measured more frequently under conditions of low perceptual load vs. high perceptual load. For example, synesthetes should report experiencing synesthetic responses more often when the triggering stimuli are presented under low load conditions.

### Perceptual crowding experiments

An additional paradigm used to examine synesthesia takes advantage of another well-known perceptual effect. A grapheme presented alone in the periphery is relatively easy to visually identify, whereas it is much more difficult to identify when it is flanked by distractor graphemes—an effect known as “crowding” (Flom et al., 1963; Bouma, 1970; Chung et al., 2001; Levi, 2008). However, identification in the flanking condition is made easier if the target grapheme is a different color than the distractors (Gheri et al., 2007).

Grapheme-color synesthetes have been tested using this paradigm in order to determine if synesthetic “colors” facilitate identification of a flanked target in the same way that regular colors do. Here, too, results have not been consistent. Ramachandran and Hubbard (2001) tested a synesthete on this task, who reported that his synesthetic “color” response was triggered, *but only on this basis* was he able to identify the flanked grapheme. In this case, the synesthetic “color” merely helped him to infer what the grapheme must have been, rather than helping him to consciously see the grapheme in the way that regular colors allow under the same conditions. Hubbard et al. (2005) also performed a crowding experiment using six synesthetes and found evidence for superior performance on the task over the control subjects in only three of the six synesthetes. Thus, the synesthetes as a whole did not exhibit significantly superior performance over controls for the task, as one might expect if synesthetic colors behaved like regular colors.

## Individual differences: is synesthesia “out there” or “all in the head”?

What are we to make of the inconsistent results that plague the literature on synesthesia? A tempting solution is to appeal to individual differences among synesthetes to explain them. One distinction that is sometimes drawn in the literature is that between “projector” and “associator” synesthetes (Smilek et al., 2001; Dixon et al., 2004; Dixon and Smilek, 2005; Ward and Mattingley, 2006; Ward et al., 2007, 2010; Jarick et al., 2011). Projector synesthetes report experiencing their synesthetic responses (e.g., color in grapheme-color synesthesia) as being located “out there in space,” whereas associator synesthetes report experiencing them as being present instead in their “mind's eye.”

There are two problems with appealing to the associator/projector distinction to explain the varying performance of synesthetes in the tasks we have just discussed: (1) Most of the motivation for positing this distinction stems from subjective reports that are difficult to interpret, and (2) the objective methods used for evaluating the associator/projector distinction fall short of establishing it.

Although there is divergence among the subjective reports of synesthetes, which is in large part the basis for the associator/projector distinction, it is difficult to determine whether this is due to differing experiences or simply varying idiolects. Indeed, some of these reports have been found to be inconsistent with one another. Edquist et al. (2006) carried out a study in which one part required that fourteen grapheme-color synesthetes respond to a questionnaire that asked them to indicate their agreement with the following: “the color is out there in space,” “the color is in my mind's eye,” or “neither.” Strikingly, the results of the questionnaire revealed seemingly conflicting reports within individual subjects. For example, two synesthetes agreed with the sentence “the color is out there in space,” but on a separate questionnaire administered subsequently, these same two synesthetes also strongly agreed with the sentence “the color is in my mind's eye.” And another three synesthetes who indicated their agreement with the sentence “the color is in my mind's eye” also agreed with the sentence “the color looks like it is on the page.” These competing responses not only highlight the need for caution in using and interpreting subjective reports for the purposes of theorizing about synesthesia and the varieties thereof, but also raise general concerns regarding the classification of individuals as synesthetes based solely on such reports.

One way to mitigate these concerns might be to more carefully choose the wording involved in questionnaires intended to probe the experiences of synesthetes, such that ambiguities are avoided. For instance, the reason some synesthetes may have agreed that their synesthetic responses are in their “mind's eye” *as well as* being “out there on the page,” is that despite experiencing their synesthetic responses as being out there on the page, they still retain the *belief* that they are not actually properties of external objects. And insofar as they hold this belief, they might be inclined to respond that the synesthetic response is in their “mind's eye.” Their seemingly inconsistent responses to the questionnaire might have been an attempt to reflect this specific stance.

Given the difficulties involved in interpreting subjective reports, the most reliable source of evidence will likely come from objective measures that corroborate these reports—at least when they are sufficiently well-understood. The main behavioral task used for these purposes is the variation of the Stroop paradigm described earlier (Stroop, 1935; MacLeod, 1991). Those who identify as projector synesthetes tend to be faster at naming synesthetic colors of letters over their veridical colors, whereas those who identify as associator synesthetes tend to be faster at naming veridical colors over synesthetic colors (Dixon et al., 2004; Ward et al., 2007). Projectors also display larger Stroop interference effects than associators when it comes to their performance on the color-naming task.

From these findings, Dixon et al. (2004) suggest that synesthetic responses in the Stroop task are more automatic for projectors than for associators because external projections of color are more difficult to ignore than internal ones. Ward et al. (2007), having replicated the results of Dixon et al., elaborate on this by offering an explanation in terms of shifting spatial frames of reference. They suggest that in order to successfully complete the task, associators must attend to the grapheme located on the computer screen and then retrieve the corresponding color from a different spatial location (i.e., their “mind's eye”). This slows them down relative to projectors, who need only attend at or near the location of the grapheme to report their synesthetic colors. As for projectors being slower at naming veridical colors than synesthetic colors, Ward et al. suggest that the real and synesthetic color in the same location leads to competition between the two.

These interpretations, while perhaps promising, require much further support before they can be used to validate the projector/associator distinction. The results might be explained equally well by those reporting to be projectors simply having stronger grapheme-color associations than those reporting to be associators, rather than a different quality in their perceptual experience, or any perceptual experience whatsoever for that matter. A more solid result comes from van Leeuwen et al. (2011), who found in an fMRI study using dynamic causal modeling that the synesthetic responses of projector grapheme-color synesthetes were driven primarily by bottom–up processes via the fusiform gyrus, while those of the associator synesthetes were generated mainly by top–down processes via the superior parietal lobe (see also Rouw and Scholte, 2007). We propose that until more reliable evidence along these lines is gathered regarding the purported projector/associator distinction, this distinction cannot help to explain away the inconsistencies in the literature on synesthesia.

Another distinction that is sometimes appealed to in characterizing individual differences in synesthesia is that between “higher” and “lower” synesthesia (Ramachandran et al., 2002)^9^. Higher synesthetes are characterized as those individuals whose synesthetic responses may be triggered in the absence of an inducing physical stimulus, just by thinking about or imagining the relevant stimulus. Lower synesthetes are those for whom the presence of the inducing physical stimulus is required in order to experience a synesthetic response. Higher synesthetes are also characterized in a second way, as those individuals for whom the conceptual properties, and not merely the sensory properties, of a physical stimulus trigger their synesthetic responses. For example, a higher grapheme-color synesthete that associates the number five with the color red might experience a red sensation in response to the written word “FIVE,” the roman numeral “V,” a cluster of five dots as on a rolling die, and the symbol “5,” all of which differ in their sensory properties but share in common the conceptual property of representing the number five (see, e.g., Ward and Sagiv, 2007). Lower synesthetes are thought not to have synesthetic experiences that are sensitive to the conceptual properties of stimuli to this degree^10^.

Some evidence for this distinction derives once again from subjective reports (e.g., Ramachandran and Hubbard, 2001). However, here too the distinction is not well-established by objective measures. Dixon et al. (2000) attempted to provide some experimental evidence for this higher category of synesthesia using a variant of the Stroop paradigm. They presented the grapheme-color synesthete, C, with arithmetic problems and asked her to calculate the solution. The solution was always a number that would typically elicit in C a report of a highly specific color experience. After calculating each sum, C then had to name the color of a patch that was either congruent or incongruent with that of the response triggered by the sum that she had just calculated. They found that C performed faster on congruent vs. incongruent trials, suggesting that, for her, having a thought about a specific number is enough to trigger the corresponding association.

Even if one accepts this study as conclusive evidence of the higher vs. lower synesthete distinction, it would not account for the inconsistent results discussed in the previous section. Higher synesthetes should consistently perform better than controls in these tasks. In addition, as with other Stroop-like tasks, C's performance could be explained without positing a perceptual synesthetic response. For example, it might be that C calculated the sum of two numbers and then rapidly or automatically recalled the color that went along with the sum rather than undergoing a perceptual experience.

Again, more evidence is required before such purported individual differences between synesthetes can be used to explain inconsistent results on perceptual tasks and classify different types of synesthetes. Thus, these various distinctions between different synesthesia subtypes may not help at this stage of inquiry.

We propose, however, that a promising way forward is to ensure that there is consistency across the various studies that examine the performance of synesthetes on different types of paradigms. For one, many of the studies do not use the same types of visual stimuli or displays. And for those that do, consistency and other control measures to rule out alternatives to synesthesia, such as superior memory, are often lacking. A standardized battery to assess synesthesia might therefore include a set of tests that are each precisely calibrated and validated to assess different defining characteristics of synesthesia. Once a standard set of criteria are used to correctly indentify and classify synesthesia, future studies will be in a better position to examine the nature of the condition.

## Conclusions

Based on this selective review that highlights some of the many challenges in synesthesia research, it is clear that the field needs more convincing evidence and better tools to assess and more fully understand the nature of this condition. As it stands, current methods leave many questions unanswered concerning the psychological kind or kinds under which synesthetic responses fall. And current assessment strategies leave too much room for error. Some of the more promising tools for better understanding and assessing synesthesia are ones that may measure it more objectively, such as those that measure brain responses during synesthetic responses using neuroimaging techniques. These tools could be used in tandem with some of the behavioral assessments and experimental paradigms we have surveyed here. Based on the current state of the neuropsychological literature on synesthesia, much further research is required before we have a clearer grasp on the underlying mechanisms involved with synesthesia.

### Conflict of interest statement

The authors declare that the research was conducted in the absence of any commercial or financial relationships that could be construed as a potential conflict of interest.

## Footnotes

^1^For a comprehensive list, as well as a discussion of the different forms that synesthesia can take, see Novich et al. (2011).

^2^In addition to automaticity, reliability, and consistency most definitions of synesthesia either state or imply that the synesthetic response occurs consciously. However, as of yet there has been no empirical work done to determine whether these responses may sometimes occur unconsciously, similar to how subliminal perception occurs (though see Mattingley et al., 2001). As such, we do not include consciousness as a feature of synesthetic associations.

^3^And there is a question, furthermore, about whether the experiences induced by drugs are appropriately classified as synesthesia. Hubbard (2007), for example, notes that congenital synesthetes have synesthetic experiences that typically involve simple colors and movements, while drug-induced hallucinations are often complex scenes or images.

^4^We use the term “association” loosely, to characterize whatever relationship holds between the stimulus trigger and the synesthetic percept. For some synesthetes, this relationship is much tighter than the term suggests, as illustrated by the following description of one synesthete: “The shapes are not distinct from hearing them—they are part of what hearing is. The vibraphone, the musical instrument, makes a round shape. Each is like a little gold ball falling. That's what the sound is; it couldn't possibly be anything else” (Cytowic, 2002, p. 69).

^5^This is not to rule out cases in which the concurrent is nonspecific or changes slightly or gradually over time (e.g., Eagleman et al., 2007).

^6^Smilek et al. (2002) and Tammet (2009) suggest that synesthetes have stronger memories due to their synesthesia, but the causal relationship may run in the opposite direction, which would be problematic for the TOG.

^7^We note that this may also be problematic in laboratory studies.

^8^Another potential issue is that web-based samples may not be representative in the way required (Skitka and Sargis, 2006). But this is a general issue in recruiting subjects for laboratory studies as well.

^9^Some have suggested that the higher vs. lower synesthete distinction maps onto the associator vs. projector synesthete distinction (Dixon et al., 2004; Dixon and Smilek, 2005) Indeed, it may be that there is only one distinction between higher/associator synesthesia and between lower/projector synesthesia. This way of distinguishing different types of synesthesias may be reasonable, but higher synesthetes sometimes have no inducing stimulus onto which to project. In these cases, higher synesthetes behave like associator synesthetes and would be classified differently. In general, it may be important to keep these pairs of synesthesia types conceptually distinct, since one is based on the nature of the inducing stimulus, and the other is based on the nature of the synesthetic experience, and higher synesthetes do, at least sometimes, respond to physical stimuli, as well as imagined or thought about stimuli. Moreover, Ward et al. (2007) administered a probing questionnaire to fourteen synesthetes that had been previously classified as either projectors or associators and found no evidence to suggest that the projectors were more likely to be lower synesthetes or that the associators were more likely to be higher synesthetes.

^10^It is important to note, however, that being a lower synesthete, in the sense just described, does not mean that the way a physical stimulus is conceptualized makes no difference to the induced synesthetic response. Ramachandran and Hubbard (2001) used ambiguous stimuli on two synesthetes that were classified as lower grapheme-color synesthetes because only Arabic numerals evoke colors for them, whereas other stimuli that represent numerosity do not. But they found that when presented with the words “THE CAT,” in which they used identical, ambiguous symbols for the “H” and the “A” (Selfridge, 1955), both synesthetes reported seeing their usual colors for “H” and “A.” The quality of their synesthetic experiences seemed to depend on whether they saw the identical symbol as an “H” or as an “A.” This finding related to ambiguous stimuli has been reported by others as well (Myles et al., 2003). Thus, there is evidence to suggest that the way that a stimulus is conceptualized makes a difference for the induced photism, even in lower synesthetes. This somewhat blurs the boundary between higher and lower synesthesia as characterized by sensitivity to conceptual properties rather than just perceptual properties, since both seem to involve such sensitivity to some degree. Consequently, it may be more reasonable to primarily characterize higher synesthesia as that which may be triggered by thought or imagination alone and lower synesthesia as that which requires the presence of physical inducing stimuli.
